# Draft Genome Sequence of *Mycobacterium tuberculosis* Strain E186hv of Beijing B0/W Lineage with Reduced Virulence

**DOI:** 10.1128/genomeA.00403-15

**Published:** 2015-05-07

**Authors:** K. V. Shur, K. M. Klimina, N. V. Zakharevich, D. A. Maslov, O. B. Bekker, M. V. Zaychikova, E. Y. Kamaev, M. A. Kravchenko, S. N. Skornyakov, Y. Zhang, V. N. Danilenko

**Affiliations:** a Laboratory of Bacterial Genetics, Vavilov Institute of General Genetics, Moscow, Russia; b Ural Research Institute for Phthisiopulmonology, Ekaterinburg, Russia; c Department of Molecular Microbiology & Immunology, Bloomberg School of Public Health, Johns Hopkins University, Baltimore, Maryland, USA

## Abstract

We report a draft genome sequence of *Mycobacterium tuberculosis* strain E186hv, belonging to the Beijing B0/W lineage and isolated from a patient from Kurgan, Russia. This clinical isolate showed a reduced virulence phenotype unusual for this lineage and resistance to isoniazid, rifampin, ethambutol, pyrazinamide, and ofloxacin. We analyzed single nucleotide polymorphisms (SNPs) associated with virulence.

## GENOME ANNOUNCEMENT

The Beijing genotype is commonly present in the Russian population and in Eurasia as a whole. This lineage is characterized by high-level virulence (1–4).

For our analysis, we chose the *Mycobacterium tuberculosis* strain E186hv, isolated from a 53-year-old HIV-negative male patient from Kurgan, Russia, with secondary diagnosed fibrocavernous tuberculosis, provided by the Ural Research Institute for Phthisiopulmonology, Ekaterinburg, Russia. This strain was resistant to isoniazid (INH), rifampin (RIF), ethambutol (EMB), pyrazinamide (PZA), and ofloxacin (FLQ) and characterized as a strain with reduced virulence in the guinea pig model (5). The genomic DNA from *Mycobacterium tuberculosis* strain E186hv was purified by use of a PREP-NA kit (DNA-technology, Russia).

Genome sequencing was carried out on a Roche 454 GS Junior instrument (Roche, Switzerland) in the Laboratory of Bacterial Genetics, Vavilov Institute of General Genetics (Moscow, Russia). A total of 176,811 reads were generated. All reads were assembled to an initial draft genome of 4,334,870 nucleotides at 24-fold coverage using the GS *De Novo* Assembler (version 3.0; Roche). The resulting draft genome sequence consists of 137 contigs (overall G+C content, 65.5%). The automatic functional annotation results were obtained using the NCBI Prokaryotic Genome Annotation Pipeline (PGAAP) (http://www.ncbi.nlm.nih.gov/genome/annotation_prok/).

The E186hv genome contains 4,027 coding sequences (CDS), 3 rRNAs, and 45 tRNAs. A total of 83 pseudogenes, 5 noncoding RNAs (ncRNAs), 1 clustered regularly interspaced short palindromic repeat (CRISPR), and 55 frameshifted genes were predicted using the PGAAP.

According to housekeeping gene analysis (6), we classified this strain as belonging to the Beijing lineage. Analysis of the *oxcA* gene showed that this strain belongs to the B0/W subline. We compared our output sequence with the DNA sequence of the highly virulent *M. tuberculosis* W-148 strain, which belongs to the B0/W cluster of the Beijing group (7). In this announcement, we focused on genes which determine drug resistance and also virulence-associated loci, including the type II toxin-antitoxin (TA) systems (8, 9), type VII secretion genes (2), genes encoding WhiB-family proteins, including WhiB7 regulon (10, 11), and Ser/Thr protein kinases (STPKs) (12–14).

In the genes, we analyzed the single nucleotide polymorphisms (SNPs) associated with drug resistance to INH, RIF, PZA, EMB, and FLQ. We found SNPs in INH resistance genes (*rv1592c*, *katG*, *fabD*, and *accD6*), in the PZA resistance gene *pncA*, in RIF resistance genes (*rpoB* and *embB*), and in the FLQ resistance gene *gyrA*. We found synonymic changes in the EMB resistance genes *embC* and *embA*. The found amino acid substitution in KatG S315T does not affect the virulence level (15, 16), and thus the reduced virulence phenotype is determined by other factors.

The comparison of sequenced DNA with the W-148 sequence revealed the presence of 709 polymorphisms. We carried out a deeper analysis of genes involved in virulence. We did not identify genetic variants of VII-type secretion genes, STPKs genes, *whiB1-7* genes, and the *whiB7* regulon genes. We found only one SNP in the TA system—antitoxin gene *vapB5* TC(167-168)CG. This mutation, as well as other SNPs associated with virulence (2, 4, 17), may impact the level of *M. tuberculosis* virulence and should be the subject for further functional analysis and validation in *M. tuberculosis* in future studies.

### Nucleotide sequence accession numbers.

This whole-genome shotgun project has been deposited at GenBank under the accession number JXAW00000000 (*Mycobacterium tuberculosis* strain E186hv). The version described in this paper is version JXAW02000000.
